# SIRT7 and hepatic lipid metabolism

**DOI:** 10.3389/fcell.2015.00001

**Published:** 2015-01-20

**Authors:** Bor Luen Tang

**Affiliations:** ^1^Department of Biochemistry, Yong Loo Lin School of Medicine, National University Health SystemSingapore, Singapore; ^2^NUS Graduate School for Integrative Sciences and Engineering, National University of SingaporeSingapore, Singapore

**Keywords:** sirtuins, Sirt7, SIRT1, lipid metabolism, mitochondria

The mammalian sirtuin family, consisting of NAD^+^-dependent class III histone/protein deacetylases, has 7 members (Greiss and Gartner, 2009). The physiological activities and pathological associations of sirtuins have been subjects of intense interest, as these are key regulators of metabolic processes that often impinge on health, aging and lifespan (Chalkiadaki and Guarente, 2012; Houtkooper et al., 2012). Perhaps the least investigated of the sirtuins is SIRT7. Like its more illustrious family member SIRT1, SIRT7 is nuclear localized. More specifically, it is enriched in the nucleolus (Ford et al., 2006; Barber et al., 2012; Ryu et al., 2014). Functionally, SIRT7 was first described by Guarente's group to be associated with active ribosomal RNA genes (or rDNA), where it interacts with RNA polymerase I and positively regulate its transcription activity (Ford et al., 2006). SIRT7 also interacts directly with the rDNA transcription factor upstream binding factor (UBF), and is responsible for resuming rDNA transcription, suppressed during cell division, upon mitotic exit (Grob et al., 2009). Proteomics analyses indicate that SIRT7 is also associated with several nucleolus-localized chromatin remodeling complexes (Tsai et al., 2012) and is involved in multiple components of pathways regulating ribosome biogenesis and protein translation (Tsai et al., 2014). Other recent findings have also implicated a role for SIRT7 in regulating aspects of cell survival in multiple tissue/organ systems. Like SIRT1, SIRT7 deacetylates p53, and in this regard appears to be cardioprotective. *Sirt7* knockout mice were shown to exhibit reduced lifespan with frequent development of heart hypertrophy and inflammatory cardiomyopathy (Vakhrusheva et al., 2008). SIRT7 also deacetylates the acetylated lysine 18 of histone H3, and together with its association with E26 transformed specific (ETS) domain containing transcription factor ELK4 and perhaps other tumor suppressor factors, appears to promote a state of malignancy (Barber et al., 2012).

A particular interesting set of recent papers (Shin et al., 2013; Ryu et al., 2014; Yoshizawa et al., 2014) reported findings on the role of SIRT7 in metabolic regulation, particularly the regulation of lipid and energy metabolism in the liver. These papers together clearly suggest a vital role for SIRT7 in hepatic lipid metabolism, which is in line with earlier data showing that the liver has the highest levels of *Sirt7* transcripts among adult mice tissues (Ford et al., 2006). However, these findings are also intriguing in the way they contradict each other. One of these papers reached an almost completely opposite conclusion on the role of SIRT7 in hepatic lipid metabolism from the other two. Even amongst the two that were in general agreement with each other, the elucidated mechanisms differ significantly.

Yoshizawa et al. (2014) studied *Sirt7* knockout (KO) mouse generated previously by Braun's group (Vakhrusheva et al., 2008) (deleting exons 4–9), which is extensively backcrossed to a C57/BL6 background. They observed that these Sirt7 KO mice were resistant to high fat diet (HFD)-induced liver steatosis (fatty liver), obesity and glucose intolerance, and showed significantly lower hepatic triglyceride (TG) content compared to wild type. The livers of HFD-fed *Sirt7* KO mice has decreased expression of TG synthesis and lipid storage genes, such as CD36 (which is crucial for fatty acid uptake), as well as the cell-death-inducing DFFA-like effector (CIDE) A and C (which functions in lipid storage and lipid droplet formation). The authors also generated liver-specific conditional knockout mice (floxed *Sirt7* crossed with albumin promoter driven Cre recombinase, or LCKO mice), and these likewise exhibited a similar phenotype of reduced hepatic TG and lipid droplets after HFD feeding. The authors found that SIRT7 positively regulates the orphan nuclear receptor testicular receptor 4 (TR4), and it activates multiple TR4 target genes to increase fatty acid uptake and TG biosynthesis and storage. This is achieved through SIRT7's interaction with an E3 ubiquitin ligase complex (the DDB1-CUL4-associated factor 1 (DCAF1)/damage-specific DNA binding protein 1 (DDB1)/cullin 4B (CUL4B) complex) (Lee and Zhou, 2007) which inhibits TR4 degradation. TR4 levels are reduced in SIRT7-deficient liver, and the steatosis-resistant phenotype could be explained by a decreased expression of lipid synthesis and storage genes.

Two other studies, however, presented findings that contradicted the above. Shin and colleagues generated another *Sirt7* knockout mouse strain (by replacing exons 4–11 with a LacZ gene), but observed instead that these mice develop liver steatosis (Shin et al., 2013). Livers of these *Sirt7* knockout mice have elevated TG levels, increased expression of inflammatory markers, and increased expression of lipogenic genes. These mice also have, paradoxically, a serum TG level that is 4-fold lower than wild type, which is likely due to reduce secretion of the TG transporter very low density glycoprotein (VLDL). Hepatocyte specific rescue of the SIRT7 deficiency with targeted adeno-associated virus 8 (AAV8)-mediated Sirt7 expression was able to reverse the phenotype. The authors noted potential binding sites for X-box binding protein 1 (XBP1), a key regulator of the unfolded protein response (UPR) in the *Sirt7* promoter, and found that ER stress upregulates *Sirt7* in a XBP1 dependent manner. They further found that ER stress underlies the development of steatosis in their *Sirt7* deficient mice, as the *Sirt7* deficient livers have increased ER stress that could be partially rescued by a small molecule chaperone, and viral mediated transgenic expression of *Sirt7*. *Sirt7* over-expression could also reverse steatosis induced by a high-fat diet (HFD). The authors provided evidence for an underlying mechanism for their observations. They showed that the protooncogene product c-Myc recruits SIRT7 at the promoters of ribosomal proteins to mediate chromatin remodeling and transcriptional repression of these, which suppresses ER stress.

In another study, Ryu and colleagues generated a different *Sirt7* knockout mouse (deleting exons 6–9), and likewise observed that these suffered from liver microvesicular steatosis, with elevated plasma TG and free fatty acids (Ryu et al., 2014). These authors, however, noted a more general metabolic defect in their *Sirt7* knockout mice that is associated with mitochondrial dysfunction, such as elevated blood lactate levels and reduced mitochondrial electron transport chain Complex I, which could be rescued by viral-mediated transgenic expression of *Sirt7*. SIRT7 levels correlated with the levels of mitochondrial ribosomal protein genes, and ChIP-seq demonstrates that SIRT7 is associated with the promoters of 232 genes encoding mitochondrial proteins. The effects of SIRT7 on mitochondrial homeostasis were apparently due to SIRT7's deacetylation of GA-binding protein (GABP) β1, a regulator of multiple nuclear-encoded mitochondrial genes. GABPβ1 deacetylation facilitates its complex formation with the ETS-domain containing GABPα, and the transcriptional activation of the GABPα/GABPβ heterotetrameric complex. SIRT7 appears therefore to be important in metabolic regulation at a more fundamental level, namely that of mitochondrial homeostasis. Hepatic lipid metabolism defects could be just one manifestation of SIRT7 deficiency.

The findings described above, while clearly pointing toward an important role for SIRT7 in hepatic lipid metabolism, is perplexing in terms of the contradicting conclusions. Differences in genetic background could on occasions contribute to phenotype differences like such, but in this case is unlikely to be a key explanation. Several points about the report of Yoshizawa and colleagues deserved to be mentioned. Firstly, the seemingly hepato-protective phenotype of the *Sirt7* knockout mice (Yoshizawa et al., 2014) itself contrasted the cardiopathic and lifespan reduction phenotype observed earlier by the Braun group (Vakhrusheva et al., 2008). While it is conceivable that SIRT7's actions are somewhat different in these two tissues, one would expect some degree of similarity in terms of systemically chronic inflammation and stress, which presumably underlie the reduced lifespan. In view of the generally unfavorable health effect of the *Sirt7* knockout (Vakhrusheva et al., 2008), analysis of how SIRT7 deficiency would affect the liver may be best done with a liver-specific conditional knockout. The authors have generated precisely such a liver-specific knockout line (LCKO) (Yoshizawa et al., 2014), but the data associated with the LCKOs were rather limited. It should also be noted that liver-specific *Sirt7* KO mice generated by Ryu et al. had microvesicular steatosis just like the global KO mice (Ryu et al., 2014).

It is conceivable that SIRT7's activity may occur in crosstalk or in tandem with other nuclear sirtuins. In this regard, SIRT1 is of particular interest, as its activity is known to mobilize stored lipids and protects against a high fat diet, albeit acting largely in adipose tissues and muscle (Pfluger et al., 2008). The results of Yoshizawa and colleagues would suggest that SIRT1 and SIRT7 have opposing effects in lipid metabolism, the former being catabolic while the later driving synthesis and storage. The Braun group had earlier presented a conference poster highlighting findings that SIRT7 may promote adipogenesis by binding to and inhibiting SIRT1 (Bober et al., 2012). The full paper has not yet appeared in print though, and the physical and functional association between these two sirtuins in the liver has remained unclear.

SIRT7 appears to have, overall, a protective and prosurvival function (Vakhrusheva et al., 2008; Kiran et al., 2014). This, together with its high levels of expression in liver, makes the notion of a beneficial function of SIRT7 in this organ somewhat more palatable. In this regard, SIRT7's regulation of mitochondrial function as elucidated by Ryu and colleagues may be particularly relevant. Again, one can draw broad similarities here between SIRT7 and SIRT1. SIRT1 has a role in mitochondrial biogenesis (Brenmoehl and Hoeflich, 2013), and its deacetylation of peroxisome proliferator-activated receptor-gamma coactivator-1α (PGC-1α) has been shown to be required for activation of mitochondrial fatty acid oxidation genes (Gerhart-Hines et al., 2007). As SIRT1 and SIRT7 activities are both regulated by the availability of NAD^+^, these sirtuins will likely be co-modulated by cellular energy status and pathological conditions that affect this status. It is also likely that Sirt1 and SIRT7 have an overlapping repertoire of substrates, with p53 amongst those already known, and others will likely be revealed soon. In terms of systemic aging and lifespan, loss of *Sirt7* (Vakhrusheva et al., 2008) resulted in a short lifespan phenotype that broadly parallel that of loss of *Sirt1* (Cheng et al., 2003) It would be somewhat incomprehensible if these two sirtuins regulate metabolic transcriptional profiles in ways that are diametrically opposite instead of complementary to each other.

Although puzzling, the discrepancies between the different findings discussed above is likely to be resolved soon with rapid advances in this field. We may soon see reports of lipid metabolism profiling of liver specific knockout and overexpression of *Sirt7* in different genetic backgrounds of metabolic dysfunction, such as the leptin mutant *ob/ob*. Resolution of these discrepancies would be important if SIRT7 is to be exploited as a therapeutic target in treatment of metabolic disorders.

## Conflict of interest statement

The author declares that the research was conducted in the absence of any commercial or financial relationships that could be construed as a potential conflict of interest.
